# Identification of the Plant Defensin (MsPDF) Gene Family in *Medicago sativa* and Analysis of Expression Patterns Under Abiotic Stress

**DOI:** 10.3390/plants14091312

**Published:** 2025-04-26

**Authors:** Meiyan Guo, Xiuhua Chen, Shuaixian Li, Jiang Tian, Wangqi Huang, Yongjun Shu

**Affiliations:** 1 Key Laboratory of Molecular Cytogenetics and Genetic Breeding of Heilongjiang Province, College of Life Science and Technology, Harbin Normal University, Harbin 150025, China or 15145577725@163.com (M.G.); hsdlsx1201@stu.hrbnu.edu.cn (S.L.); 2 International Agriculture Research Institute, Yunnan Academy of Agricultural Sciences, Kunming 650200, China; cxh@yaas.org.cn (X.C.); 18213502005@163.com (J.T.); 3 National Engineering Research Center for Ornamental Horticulture, Yunnan Flower Breeding Key Laboratory, Flower Research Institute, Yunnan Academy of Agricultural Sciences, Kunming 650200, China

**Keywords:** plant defensin, alfalfa, cold stress, expression, protein structure prediction

## Abstract

*Medicago sativa* L. (alfalfa) is a major forage crop due to its high yield and stress resilience. However, its growth and productivity are often compromised by abiotic stresses, including cold, drought, and salinity. The plant defensin (PDF) gene family plays a crucial role in resistance to abiotic stress. In this study, a total of 11 MsPDF gene family members were identified in the alfalfa genome and classified into three groups. Phylogenetic and conserved motif analyses revealed that the MsPDF genes are highly conserved. Promoter analysis, gene regulatory network analysis (GRN), and gene ontology (GO)-enrichment analyses were used to infer the potential functions of *MsPDF* genes. The results showed that the gene actively responds to abiotic stress, participates in phytohormonal responses, and regulates plant growth and development through gene interactions. Transcriptome and qRT-PCR analyses showed that most of the *MsPDF* genes were significantly up-regulated under cold, drought, and salinity stresses. Among them, the *MsPDF03* exhibited superior performance under cold stress. The *MsPDF04*, *MsPDF08*, and *MsPDF09* genes were able to respond positively to drought and salt stresses. Finally, the monomeric, dimeric, and tetrameric structures of the proteins encoded by the *MsPDF* genes were predicted using AlphaFold 2 software. This study lays the foundation for the identification and evolutionary relationship analysis of the MsPDF gene family, and provides a new reference for subsequent research on abiotic stress resistance.

## 1. Introduction

Plant defensins (PDF) are basic, cysteine-rich cationic peptides with antimicrobial activity. They are 45–54 amino acids in length, and the encoded proteins are approximately 5 kDa [1]. Studies have indicated that plant defensins are crucial components of the innate defense system in plants [2]. Nuclear magnetic resonance (NMR) analysis of the solution structures of the radish defensin RsAFP1 [3], the mung bean defensin VrD1 [4], and pea defensin PsD1 [5] showed that these molecules adopt a compact, globular fold. Its tertiary structure comprises an α-helix (Asn18–Leu28) and three antiparallel β-sheets (β1: Lys2–Arg6; β2: His33–Tyr38; β3: His43–Pro50), forming a characteristic β-sheet fold. The eight conserved cysteine residues form four pairs of disulfide bonds with a connectivity of Cys1–Cys8, Cys2–Cys5, Cys3–Cys6, and Cys4–Cys7. Among these, the Cys1–Cys8 and Cys2–Cys5 pairs exhibit the highest degree of conservation. The central strand of the β-sheet is connected to the α-helix through four disulfide bridges, collectively forming a characteristic cysteine stabilized αβ (CSαβ) structural motif. [6].

In 1966, animal defensins were first characterized in the neutrophils of mice and guinea pigs, where these peptides were initially designated as “lysosomal cationic proteins” [7]. In 1985, Robert et al. isolated granule-rich sediments from human neutrophils, which contained three small antibiotic peptides. These peptides were later designated as defensins, marking the first formal definition of the defensin family [8]. In 1989, insect defensins had been isolated and purified [9]. During the 1990s, plant defensins were isolated from barley and wheat and named γ1-P and γ1-H, respectively [10,11]. In 1992, two novel antifungal peptides (each approximately 5 kDa) were isolated from radish seeds; both proteins exhibited a broad spectrum of antifungal activity [12]. This protein was initially named γ-thionine [13] and was renamed plant defensin in 1995 [14]. Subsequent studies characterized the *plant defensin* genes in diverse species, including *Arabidopsis thaliana* [15], *Pisum sativum* [16], *Sorghum bicolor L.* [17], *Medicago truncatula* [18], *Nicotiana tabacum* [19], *Oryza sativa L.* [20], *Zea mays* L. [21], and *Solanum tuberosum* L. [22]. This suggests that plant defensins are widespread throughout the plant kingdom.

During their growth and development, plants encounter various abiotic stresses, such as cold, drought and salinity stresses, which can significantly reduce crop yields and lead to substantial economic losses. In order to overcome these environmental stresses, plants have evolved a number of protective mechanisms to protect themselves from adverse conditions [23]. In recent years, knockout and overexpression studies of *plant defensin* genes in diverse plants have shown their intrinsic resistance and broad-spectrum antimicrobial activity at low concentrations. This broad-spectrum antimicrobial activity has been attributed to their ability to inhibit a wide range of pathogens, including bacteria [24], fungi [25], and viruses [26], suggesting their candidacy for enhancing disease resistance in crops. Plant defensins also inhibit ion channels for Na^+^ [27], K^+^, and Ca^2+^ [28]. For example, the defensin of *AtPDF2.3* is able to block the Kv1.2 and Kv1.6 potassium channels [29], thereby disrupting the signaling pathways. Furthermore, plant defensins do not directly bind to the membrane phospholipids but interact with complex lipids, such as sphingolipids of fungal cell membranes. This interaction alters the permeability and structural integrity of the membrane, ultimately leading to fungal cell death. They also play a crucial protective role by safeguarding crops during early developmental stages and bud formation against pathogen attacks [30]. Plant defensins possess diverse biological functions and great application prospects.

*Medicago sativa* L. (alfalfa) is one of the important leguminous forage crops. Characterized by its high yield, high nutritional value, and excellent palatability, it is one of the most important forage crops and is widely recognized as the “King of Forage”. Alfalfa, widely distributed in north and northeast China, as well as other regions, is highly susceptible to cold [31], drought [32], and salinity stresses [33] during its seedling stage.

The classifications and functions of the plant defensin gene family have been well reported in Arabidopsis, but they have not been studied in alfalfa. In this study, we performed a genome-wide identification of the PDF gene family in alfalfa using bioinformatics methods, followed by phylogenetic analysis, conserved motif analysis, cis-acting element analysis, genetic regulatory network analysis, and gene ontology annotation analysis. We also analyzed tissue expression patterns and used quantitative real-time PCR (qRT-PCR) to analyze the expression levels of *MsPDFs* under cold, drought, and salinity stresses. In addition, we have used AlphaFold 2 to predict the protein structures of members of the MsPDF gene family, including monomeric, dimeric, and tetrameric structures.

## 2. Results

### 2.1. Identification and Characterization of PDF Gene Family Members in Alfalfa

To identify members of the alfalfa PDF gene family, the homologous protein sequences of PDF in the alfalfa genome were identified by a BLAST (version 2.9.0+) and HMM search, using the Arabidopsis PDF protein sequences as a reference. After filtering sequences with incomplete domain and redundant sequences, we identified 11 *MsPDF* genes, designated as *MsPDF01*–*MsPDF11*, as shown in Table 1. The encoded proteins ranged from 73 to 99 amino acids, with *MsPDF03* being the longest (99 aa), and *MsPDF06*, *MsPDF08* the shortest (73 aa), indicating significant variation among family members.

### 2.2. Phylogenetic Analysis of the PDF Gene Family in Alfalfa, Arabidopsis, and Rice

**Figure 1:**
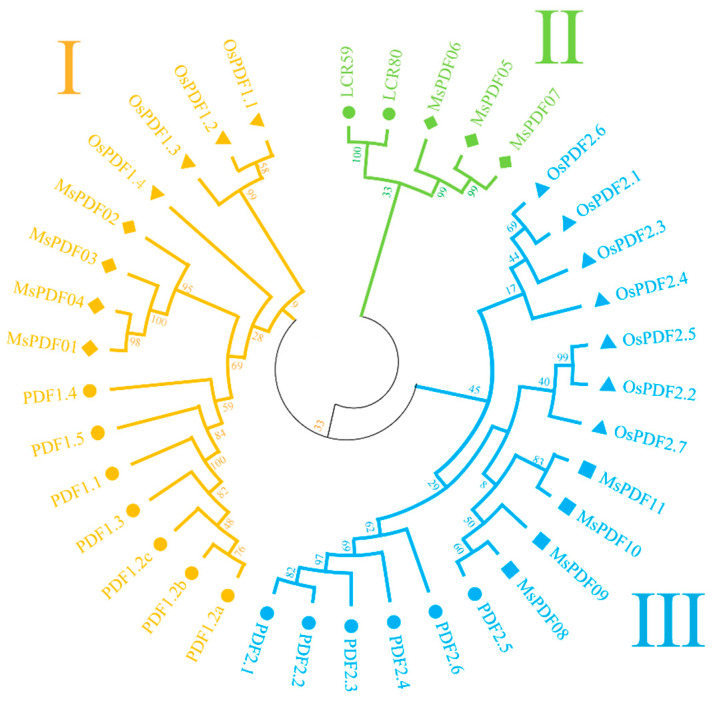
Phylogenetic analysis of PDF gene family members in alfalfa, Arabidopsis, and rice. The evolutionary tree was constructed using the neighbor-joining (NJ) method with 1000 bootstrap iteration. The *PDF* genes were classified into three subfamilies: Subfamily I (yellow), Subfamily II (green), and Subfamily III (blue). Gene members from alfalfa, Arabidopsis, and rice are symbolized by filled diamonds, circles, and triangles, respectively.

The phylogenetic tree was constructed using the neighbor-joining (NJ) method to analyze evolutionary relationships among 11 alfalfa, 15 Arabidopsis, and 11 rice *PDF* genes (Figure 1). The phylogenetic tree shows the evolutionary relationships and groupings of the MsPDF gene family members.

These genes were classified into three subfamilies based on their homology with Arabidopsis *PDF* genes, named Group I–III. The numbers of the members of different groups are shown in Figure 1. Group II has the least number of members with only three genes, and has been evolutionarily conserved, indicating its potentially important role in plant growth and development. Three orthologous gene pairs were identified: *MsPDF01* and *MsPDF04*, *MsPDF05* and *MsPDF07*, and *MsPDF10* and *MsPDF11*.

In addition, Group I contains seven members of Arabidopsis *PDF* genes and four members of rice *PDF* genes; Group II contains two members of *AtPDFs*, and Group III contains six members of *AtPDFs* and seven members of *OsPDF* genes.

### 2.3. Analysis of Conserved Motifs in the PDF Gene Family in Alfalfa

**Figure 2:**
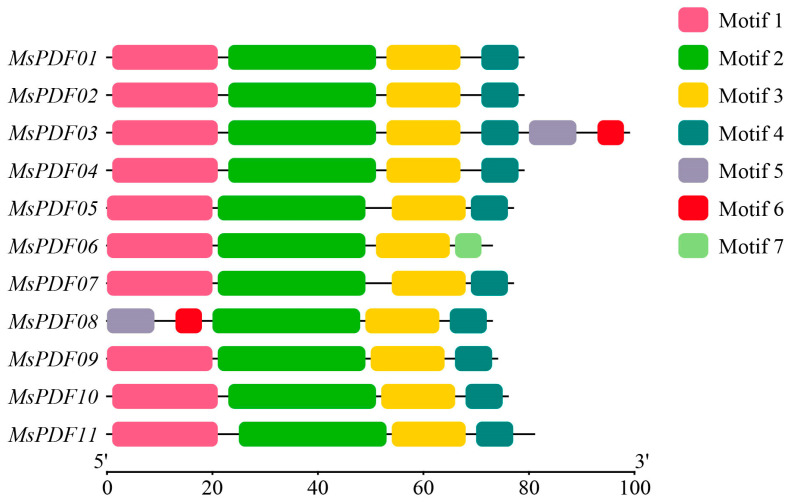
Distribution of conserved motifs in protein sequences encoded by *MsPDF* genes. Different colored rectangles represent different motifs.

Using MEME (version 4.8.1) software, the conserved motifs of alfalfa MsPDF genes were analyzed and their chromosomal distributions mapped (Figure 2). The protein sequences encoded by the 11 *MsPDF* genes contain seven conserved motifs. All identified MsPDF genes have conserved structural domains of the PDF family. All genes contained motif 2 and motif 3; motif 4 was present in all members except MsPDF06; while motif 1 were absent only in MsPDF08; motifs 5 and 6 were exclusively observed in MsPDF03 and MsPDF08. The sequence structures of the different MsPDF proteins were highly similar, with all genes having a motif order of 1-2-3-4, except for MsPDF03, MsPDF06, and MsPDF08.

In addition, we characterized the composition of conserved motifs in MsPDF proteins (Figure S1), and details of the seven conserved motifs can be found in it. The motif composition of MsPDF proteins sequences showed significant variation. Analyzing the composition of the seven conserved motifs, we found that motif 1, motif 2, motif 4, and motif 7 contained the characteristic residues of “Gamma-thionin”, which suggests their related functions. Additionally, all MsPDF protein sequences contained motif 2, which infers that this domain plays an important biological role in the *MsPDF* genes.

### 2.4. Analysis of Cis-Acting Elements in the Promoter Region of the PDF Gene Family in Alfalfa

**Figure 3:**
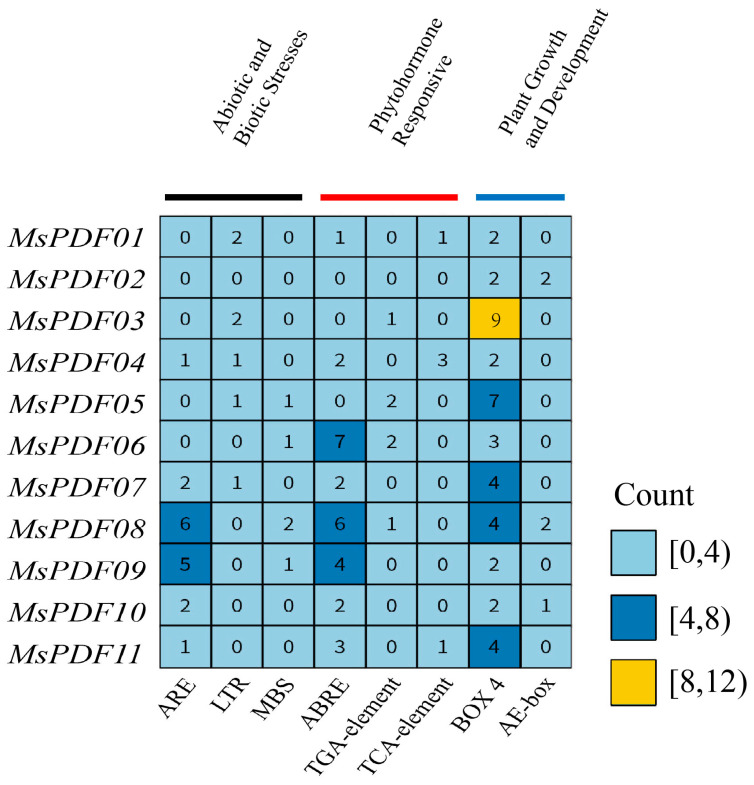
Cis-acting elements in the 2000bp promoter region upstream of the *MsPDF* genes. These elements are divided into three groups based on their abundance, marked light blue, dark blue, and yellow.

To analyze the expression and stress response patterns of PDF gene family members in alfalfa, the 2000bp upstream sequence of the *MsPDFs* was extracted as a promoter region, and cis-acting elements within this region were analyzed using the Plant CARE database. The eight elements with the highest frequency of occurrence were selected and categorized into three groups as follows (Figure 3): abiotic and biotic stress-related elements, including anaerobic response elements (ARE), low-temperature response elements (LTR), and MYB binding sites (MBS); plant hormone response elements, including abscisic acid response elements (ABRE-element), TGA transcription factor binding sites (TCA-element) and salicylic acid response elements (TCA-element); and plant growth and development-related elements, including two light-responsive elements (BOX 4, AE-box). The results showed that anaerobic response elements, abscisic acid response elements, and light-responsive element BOX 4 were predominant in *MsPDF* genes. Specifically, *MsPDF05* contained seven light responsive elements, second only to *MsPDF03*, while anaerobic response elements and ABRE elements were significantly enriched in *MsPDF08* and *MsPDF09*. Notably, *MsPDF04* contained three TCA-elements, significantly higher than other genes. Differential cis-acting elements’ distribution among genes suggests functional divergence and distinct regulatory roles in plant growth and development.

### 2.5. Regulatory Network and GO Analysis of MsPDFs Genes in Alfalfa

**Figure 4:**
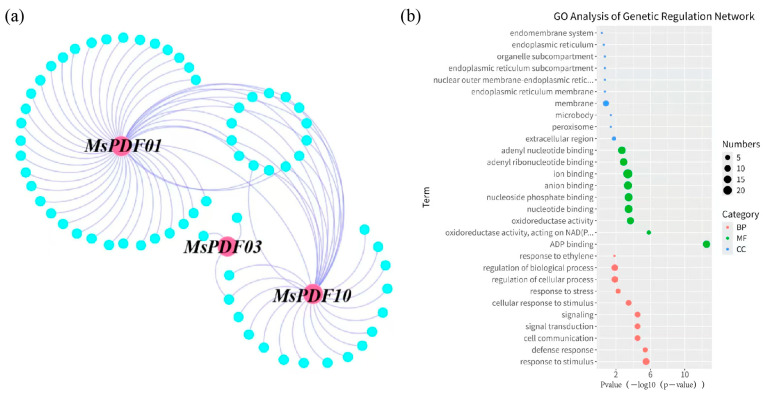
Gene regulatory network and GO enrichment analysis of the alfalfa *PDF* genes (**a**) Interaction network between *MsPDF* genes and other genes in alfalfa. Cyan nodes indicate other alfalfa genes, pink nodes indicate *MsPDF* genes, and the connecting lines between nodes indicate genes interactions. (**b**) GO enrichment analysis of *MsPDF* genes. Blue dots represent GO terms from biological process (BP), green dots for molecular function (MF), and red dots for cellular component (CC). The dot size reflects the number of enriched genes in each GO term. The X-axis shows the −log10 transformed *p*-value from the topGO enrichment analysis, while the Y-axis lists specific GO terms.

Gene regulatory network analysis identified key regulatory genes in alfalfa, and the results were visualized using Cytoscape (v3.10.0) software (Figure 4a). The *MsPDF* gene regulatory network consists of 68 genes and 80 interaction relationships. Members of the MsPDF gene family can interact with many other alfalfa genes to regulate growth and development. Among them, *MsPDF01* interacts with 47 genes (Table S2), suggesting that it plays an important role in the regulatory network; it is closely followed by *MsPDF10* which interacts with 31 genes, whereas *MsPDF03* genes showed minimal connectivity (3 interactions). In addition, *MsPDF01* and *MsPDF10* can interact with a shared set of 12 alfalfa genes, as shown by Figure 4a.

Gene ontology enrichment analysis was utilized to analyze the potential functions of *MsPDF* genes (Figure 4b). We enriched the *PDF* genes for biological processes (BP), molecular functions (MF), and cellular components (CC). The results show that *MsPDFs* genes are widely distributed in cell and membrane structures. In addition, *MsPDF* genes are able to bind to nucleotide phosphate and ions and exhibit certain oxidoreductase activities, further demonstrating the regulatory mechanism of *MsPDF* genes. These results demonstrate that *MsPDF* genes widely regulate diverse biological events.

### 2.6. Expression Analysis of PDF Genes in Alfalfa Under Abiotic Stress

To further investigate *MsPDF* gene responses to abiotic stress, transcriptome datasets were downloaded from the NCBI database. The accession numbers are PRJNA780579 for cold stress and PRJNA667169 for drought and salinity stresses. Gene expression heatmaps were visualized using R (v4.4.2) software.

**Figure 5:**
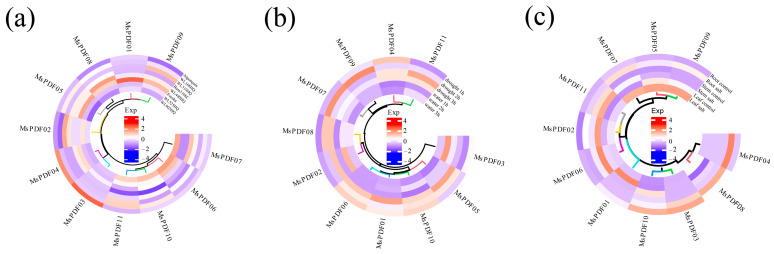
Expression profiles of *MsPDF* genes under different abiotic stresses. (**a**) Cold stress: eight alfalfa varieties were applied for 3 h at 4 °C treatment. Subsequently, total RNA of leaf was extracted and RNA-seq was performed by BGI-Seq500 model. (**b**) Drought stress: expression analysis of *MsPDF* genes in roots, stems, and leaves of the Sanarac alfalfa variety under drought stress. (**c**) Salinity stress: expression pattern of *MsPDF* genes in three tissues of Wilson alfalfa variety under salinity stress. Each experiment was repeated three times. Salmon (version 0.12.0) software was employed to measure the average expression levels (FPKM values) and the R (version 4.4.2) platform was employed to visualize the results in a hierarchical cluster analysis.

The results demonstrated that most MsPDF genes were up-regulated under cold stress, notably *MsPDF03* (Figure 5a). This suggests that *MsPDF* genes play an important role in resistance to cold stress. The expression levels of all *MsPDF* genes were significantly up-regulated compared with the control. *MsPDF07* and *MsPDF09* genes exhibited high expression under drought stress. Salt treatment was applied to alfalfa roots, stems, and leaves, and the overall expression of *MsPDF* genes was up-regulated in response to salinity stress treatments, and were shown to be tissue-specific (Figure 5c). Among them, *MsPDF04* and *MsPDF06* showed marked up-regulation compared with the control under salinity stress. Drawing from the above results, *MsPDF* genes are able to respond positively to abiotic stress.

### 2.7. Expression Analysis of MsPDF Genes Under Cold, Drought, and Salinity Stress

**Figure 6:**
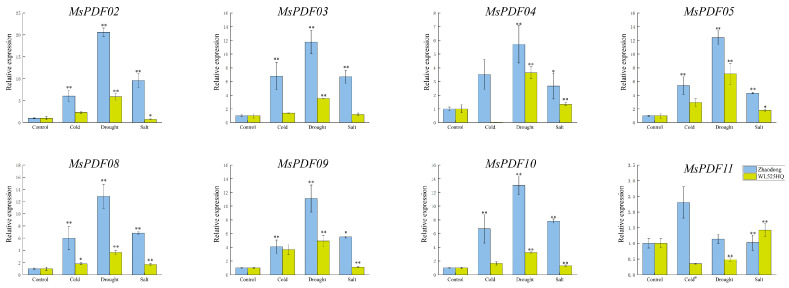
Expression analysis of the *MsPDF* genes under cold, drought, and salinity stresses. The X-axis represents the experimental groups comprising untreated controls and three stress treatments (cold, drought, and salinity). The Y-axis represents the relative expression of *MsPDF* genes, which was normalized to 1 for the control group. Asterisks represent significant differences between different groups (* *p* ≤ 0.05, ** *p* ≤ 0.01) using *t*-test for comparison of groups. Expression data were calculated using the 2^−ΔΔCT^ method.

To thoroughly investigate the *MsPDF* genes’ responses to abiotic stress and validate transcriptome data, alfalfa plants were subjected to low temperatures at 4 °C, 20% PEG-6000 solution, and 100 mM NaCl solution to simulate cold, drought, and salinity stresses. Each treatment was replicated three times. The gene expression levels were analyzed using qRT-PCR, and the results are shown in Figure 6.

Eight *MsPDF* genes were selected for qRT-PCR analysis. The results show that the expression levels of the all the *MsPDF* genes were significantly up-regulated under cold stress, except the *MsPDF04* gene. This is consistent with the results of RNA-seq; these findings demonstrate that alfalfa *MsPDF* genes actively respond to cold stress

In addition, the expression level of the *MsPDF* genes was also significantly higher than the control under drought and salinity stresses. For example, the expression of the *MsPDF04*, *MsPDF08*, *MsPDF09*, *MsPDF10*, and *MsPDF11* genes were significantly increased under salinity stress compared with the control, which indicated that *MsPDF* genes play an important role in response to abiotic stress. This conclusion further supports the RNA-seq results.

### 2.8. Structure Prediction of Proteins Encoded by the MsPDF Genes

**Figure 7:**
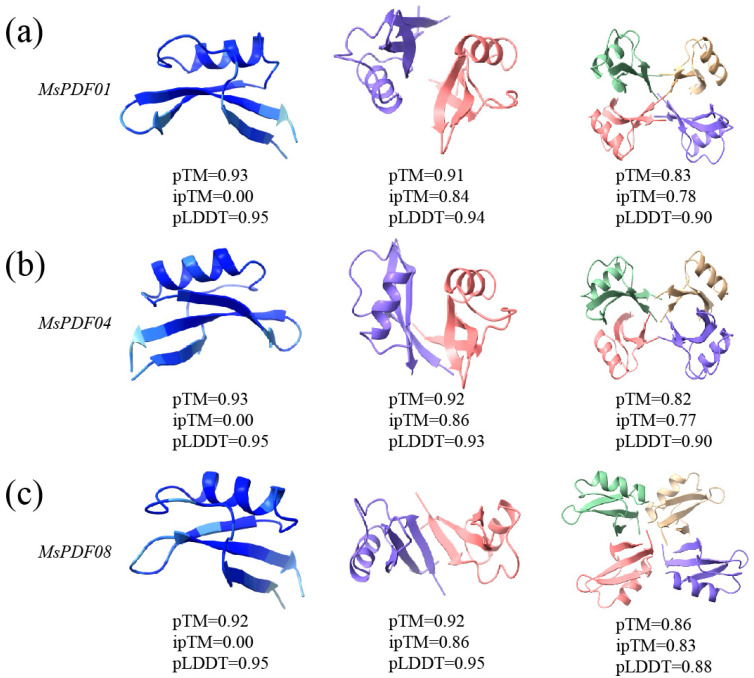
Structural prediction of monomer, dimer, and tetramer in MsPDF proteins. (**a**) Structures of the protein encoded by the *MsPDF01* gene. (**b**) Structures of proteins encoded by the *MsPDF04* gene. (**c**) Structures of the protein encoded by the *MsPDF08* gene. Monomers were color coded using the AlphaFold scheme. In the dimeric structure, the two subunits are represented in pink and purple. The tetrameric structure distinguishes its four subunits in green, yellow, pink, and purple. Prediction confidence was assessed using pTM and ipTM scores. The pLDDT score was used to assess the model prediction accuracy for each residue position. The results were visualized using ChimeraX (version 1.9) software.

In order to better analyze the function of *MsPDF* genes and analyze the mechanism of action, AlphaFold 2 software was used to predict the tertiary and quaternary structures of the proteins, which were encoded by *MsPDF01*, *MsPDF04i*, and *MsPDF08* genes. The results are shown in Figure 7. The cysteine content of these proteins was 16–17%. In addition, the secondary structure prediction showed that these proteins were mainly composed of α-helices, β-sheets, and random curls. These proteins may respond to abiotic stress through dynamic structure changes.

The predicted template modelling (pTM) score was used to evaluate the overall prediction confidence, with a pTM > 0.5 indicating reliability. The interface prediction template modelling (ipTM) score served to evaluate the accuracy of protein–protein complex prediction, with an ipTM > 0.8 indicating reliability. The ipTM scores of the dimeric structure prediction all exceeded 0.8. The tetrameric structure of the MsPDF08 protein reached 0.83, precisely mapping each protein subunit. Furthermore, the predicted local distance difference test (pLDDT) scores of the monomeric and dimeric structures exceeded 0.90 and the tetrameric structure surpassed 0.85, which demonstrated high confidence predictions.

In conclusion, the MsPDF proteins form stable dimeric and tetrameric structures, which may participate in complex biological processes through the mechanism of multimerization regulation. This structural analysis provides a framework for clarifying the molecular mechanisms of *MsPDF* genes mediating abiotic stress responses.

## 3. Discussion

Alfalfa is a perennial herbaceous plant with high nutritional value for herbivores. Research indicates that *PDF* genes are involved in responses to diverse abiotic stresses; therefore, identifying and characterizing the MsPDF gene family and validating their potential functions is critical.

In this study, we employed BLAST alignment and domain searching to identify members of the MsPDF gene family in alfalfa, using the protein sequences of *AtPDF* genes as a reference. Previous studies have revealed that Arabidopsis has 15 *PDF* gene family members [34], *Triticum aestivum* L. has 73 members, corn contains 15 members, rice has 11 members [35], *Vitis vinifera* L. possesses 79 *DEFL* gene family members [36], and they are widely distributed in various plant species. Phylogenetic trees of alfalfa, Arabidopsis, and rice were constructed using the neighbor-joining method. These genes were classified into three subfamilies based on their homology with Arabidopsis *PDF* genes. Each group contains both At*PDF* and *MsPDF* genes, indicating high evolutionary conservation between alfalfa and Arabidopsis. However, the *OsPDF* genes were distributed in Group I and Group III, which indicated evolutionary distinctions between rice, alfalfa, and Arabidopsis. Furthermore, phylogenetic analysis revealed an orthologous gene pair between alfalfa and Arabidopsis: *MsPDF08* and *PDF2.5* (Figure 1). This finding demonstrates that alfalfa exhibits a closer phylogenetic relationship with *AtPDFs*.

Conserved motif analysis showed that the MsPDF protein sequence contains seven conserved motifs. The arrangement order of these motifs is basically consistent in the protein sequences (Figure 2), indicating a high degree of evolutionary conservation in these genes. Divergence in motif alignment between MsPDF proteins may lead to functional differences. Surprisingly, each protein sequence contained motif 2 and motif 3, suggesting that all genes are capable of performing this function and that the presence of these motifs is essential for alfalfa growth and reproduction. In addition, an analysis of motif composition showed that different motifs consisted of different amino acids (Figure S1). However, most motifs contained cysteine residues characteristic of the PDF gene family, which is consistent with previous studies.

During growth, plants are affected by a variety of stresses. Abscisic acid (ABA) signaling pathways are involved in biotic and abiotic stress responses in plants [37]. For example, wheat treated with ABA exhibited the protection of tapetum against fungal infection [38]. The expression of the ABA response protein ABR17 in pea enhanced defense-related transcripts in Arabidopsis [39]. The ABA response element (ABRE) also acts as a positive regulator of ABA signaling under saline and drought conditions [40]. An analysis of the 2000bp region upstream of the *MsPDF* genes showed a high abundance of ABRE elements. This finding implies that *MsPDF* genes likely participate in ABA-mediated osmotic stress response pathways, thereby enhancing tolerance to drought and salinity stresses. The ARE element is considered to be an ethylene reaction element. Ethylene is a hormone in plants that not only promotes fruit maturation but also participates in the response to abiotic stresses in plants. In Arabidopsis, ERF1 enhances plant stress tolerance by integrating ethylene and ABA signaling pathways under salinity and drought conditions [41]. In addition, three TCA elements were identified in the *MsPDF04* gene. Salicylic acid (SA) is a simple phenolic compound that acts as a plant growth regulator and synergizes with other hormones to combat heavy metal stress [42]. In addition, the SA-mediated signaling pathway interacts with endophytic fungi to reduce cytoplasmic cadmium (Cd) accumulation, which is capable of maintaining normal cellular functions in rice [43]. SA is a key signaling molecule that enhances plant tolerance to abiotic stress by regulating photosynthesis, the accumulation of metabolites, and redox homeostasis [44]. Based on this, it is suggested that the *MsPDF* genes play an important role in resistance to abiotic stress.

Three of the eleven members of the MsPDF gene family are involved in the gene regulatory network (*MsPDF01*, *MsPDF03*, and *MsPDF10)*. These genes interact with other genes in alfalfa to regulate plant growth and development (Table S2). Previous studies have shown that the *PDF* genes can interact with the cell membrane of G^-^ bacteria for antibacterial effects [45], consistent with this study. In addition, it is well known that metallic elements play a crucial role in plant stress resistance. For example, potassium (K) can promote the activation of enzymes and enhance the processes of photosynthesis and sugar metabolism, thereby increasing the resistance of plants to cold, drought, and salinity stresses. Furthermore, potassium is involved in the detoxification of reactive oxygen species (ROS) [46]. Calcium (Ca) is found in plant cell walls and acts as a second messenger in signal transduction [47]. CRLK1 kinase plays a bridging role in calcium/calmodulin signaling and cold signaling [48]. The GO analysis results revealed that the *MsPDF* genes were significantly enriched in ion binding sites, implicating their important role in resistance to abiotic stresses.

Plants are subjected to a variety of abiotic stresses that negatively affect them during growth. Through continuous evolution, plants have developed numerous response mechanisms to cope with these stresses. A study in Arabidopsis showed that the transcriptional activity of the *CAT* genes was significantly enhanced under cold stress treatment [49]. CAT is a major scavenger of peroxisomal H_2_O_2_ and is capable of degrading H_2_O_2_ to H_2_O and O_2_ [50]. In addition, CA-treated tobacco was also able to induce the accumulation of H_2_O_2_, as well as increase the activities of superoxide dismutase (SOD), peroxidase (POD), and catalase (CAT), which then improved the cold tolerance of tobacco [51]. In the same way, the tobacco *WsOsm* gene enhances plant resistance through its interaction with the defensin WsDF protein under salinity, drought, and cold stresses [52]. *AnWRKY29* enhances plant resistance to salinity and drought tolerance by directly binding to the promoter of the defensin genes, thereby activating its transcription [53]. The Laminarin polysaccharide has been shown to activate the expression of defensin-like genes, thereby responding to drought and salinity stresses actively [54].

In this study, the transcriptome data of alfalfa under different abiotic stresses were analyzed to investigate the stress resistance of the *MsPDF* genes (Figure 5). The results showed that the expression of most *MsPDF* genes was up-regulated under cold stress, especially *MsPDF03*. Previous studies have demonstrated that H_2_O_2_ can reduce the levels of malondialdehyde (MDA) and the cold injury index (CI) by activate the antioxidant system, thereby reduce cold injury in cucumber seedlings [55].

Under drought stress, the expression of *MsPDF07* and *MsPDF09* was most significantly up-regulated. *MsPDF04* and *MsPDF06* exhibited the strongest resistance to salinity stress. The above results show that the *PDF* genes play a particularly important role in root growth and development. Further validation of the results using qRT-PCR results demonstrate that the majority of *MsPDF* genes were significantly up-regulated under abiotic stresses.

*Plant defensin* genes are widely recognized for their antimicrobial properties, and significant progress has been made in enhancing salinity and drought tolerance. However, relatively few studies have been conducted on their cold resistance. In this study, we conducted a systematic investigation of the cold resistance mediated by the *MsPDF* genes, which will provide a theoretical reference for the future clarification of the cold tolerance mechanism of the *MsPDF* genes.

## 4. Materials and Methods

### 4.1. Identification and Characterization of MsPDF Genes

Protein sequences of 15 members of the PDF gene family in Arabidopsis were downloaded from the TAIR database (TAIR, https://www.arabidopsis.org (accessed on 7 January 2025)) [34], and *Medicago sativa* protein (PRJNA540215) from the National Centre for Biotechnology Information database (NCBI, https://www.ncbi.nlm.nih.gov/ (accessed on 15 February 2025)) [56]. We performed BLAST alignment of alfalfa protein sequences using protein sequences of AtPDFs gene family members as query sequences. The threshold e-value was set to 1 × 10^−^⁵ and the maximum number of target sequences was set to 1 to eliminate redundancy. In this way, similar or identical protein sequences of Arabidopsis and alfalfa were initially identified.

The Hidden Markov Model (HMM) file for the PDF domain (PF00304) was downloaded from the Pfam database (https://pfam.xfam.org/search (accessed on 15 February 2025)) [57]. HMMER search was performed using the HMMER (version 3.3) software, and the e-value was set to 0.01 to further eliminate members with incomplete domains. We combined previous BLAST results to obtain intersections and manually removed duplicate sequences. From the alfalfa genome annotation file, the chromosomal location, protein length, and number of introns of the *PDF* genes were extracted. Based on the annotation information, the genes were categorized into groups for further analysis.

### 4.2. Phylogenetic Analysis of the MsPDFs, AtPDFs and OsPDFs

In order to analyze the evolutionary relationships among the members of the MsPDF gene family, the protein file of the *OsPDF* genes was downloaded from the NCBI database [35]. The multiple sequence alignment of PDF protein sequences from alfalfa, Arabidopsis, and rice was performed using MUSCLE software (version 5.1.0) [58]. The phylogenetic tree was constructed by the neighbor-joining (NJ) method using MEGA11.0 software with bootstrap replicates set to 1000 [59]. The bootstrap value was used to evaluate the reliability of branch divisions in phylogenetic trees, with higher values indicating greater branch support [60].

### 4.3. Analysis of Conserved Motifs and Gene Structures of MsPDF Genes

To explore conserved motifs and gene structures in the alfalfa PDF protein sequences, motif analysis was performed using MEME (version 4.8.1) Suite [61]. The parameters were set as follows: each position in the sequence is independent; each motif can appear at most once in each protein sequence; the maximum number of conserved motifs to be recognized is set to 10; and the motif width ranges from 6 to 50 amino acids. Results were visualized using bootstrap TBtools (version 0.6735) software [62].

### 4.4. Analysis of Cis-Acting Elements in the Promoters of MsPDF Genes

The 2000bp upstream region of the *Medicago sativa* genomic sequences was extracted as the promoter region. The extracted sequence information was submitted to the Plant CARE database (http://bioinformatics.psb.ugent.be/webtools/plantcare/html/ (accessed on 17 February 2025) to analyze cis-acting elements of the *MsPDF* genes.

### 4.5. Gene Regulatory Network Analysis and Gene Ontology (GO) Annotation Analysis of MsPDF Genes

The gene regulatory network (GRN) information of Arabidopsis was downloaded from the AraNet database (https://www.inetbio.org/aranet/downloadnetwork.php (accessed on 17 February 2025) [63]. The reciprocal BLAST alignment was performed between all protein sequences of Arabidopsis and alfalfa. The threshold e-value was set to 1 × 10^−^⁵, and the highest scoring hits were identified as homologous genes in Arabidopsis or alfalfa and homology was established. Homologous gene pairs were generated from the results of BLAST alignment. Based on the gene regulatory network of Arabidopsis, the generated homology relationships were used to construct the gene regulatory network of alfalfa. Network visualization was performed using Cytoscape (v3.10.0) software [64], and emphasis was particularly placed on highlighting the subnetwork of *MsPDF* genes.

To analyze the molecular functions, involved biological processes, and cellular components of *MsPDF* genes in the subnetwork, The gene ontology (GO) enrichment analysis of the subnetwork was performed using the topGO (version 2.38.31) software [65]. A threshold of 0.05 was set. The top 10 significantly enriched terms in each category were selected. Enrichment analysis was performed using Fisher’s exact test, and the results were characterized accordingly.

### 4.6. Expression Analysis of MsPDF Genes in Response to Abiotic Stresses

To investigate the response of alfalfa to cold stress, eight alfalfa varieties were selected as the research subjects for cold stress (PRJNA780579) [66] and total RNA was extracted using the RNeasy Plant Mini Kit (Qiagen, Valencia, CA, USA) following the manufacturer’s instructions. RNA samples were sent to BGI-Shenzhen Co., Ltd. (Shenzhen, China), and RNA-seq was performed on the BGI-Seq500 platform according to the manufacturer’s instructions to generate 150bp paired end reads. Roots, stems, and leaves of the Sanarac variety were used to study the response to salinity stress (PRJNA667169) [67], while the Wilson variety was investigated to study the response to drought stress (PRJNA667169) [67]. RNA-seq data were downloaded from the NCBI database. Using Salmon (version 0.12.0) software, RNA-seq data were aligned with transcript sequences from the alfalfa genome. Gene expression levels indicated by FPKM values were log2 transformed. Finally, the obtained data were clustered and visualized by the R platform (version 4.4.2), and expression heatmaps were generated.

### 4.7. qRT-PCR Analysis

To study the response of *MsPDF* genes to abiotic stresses, Zhao Dong and WL525HQ varieties were selected for real-time quantitative PCR (qRT-PCR) analysis. Firstly, the seeds were vernalized at 4 °C for 3 d and subsequently germinated. The seedlings were transplanted into a 3:1 (*v*/*v*) substrate mixture of perlite and sand. The growing environment was maintained at a constant temperature of 24 °C, and 16 hours of light and 8 hours of dark for six weeks. Seedlings were divided into four groups: the first group was treated with low temperature treatment at 4 °C, the second group was treated with 20% PEG-6000 solution to simulate drought stress, the third group was treated with 100 mM NaCl nutrient solution to simulate salinity stress, and the fourth group served as a control group, which was rinsed with conventional nutrient solution without any additional treatment. The plants in each group were treated for 1 hour, 3 hours, and 4 hours, and approximately 1 g of healthy leaf samples was collected at each time point. The leaves were mixed and immediately frozen in liquid nitrogen and stored at −80 °C for subsequent RNA extraction.

Total RNA was extracted from the four groups using the RNA pure Plant Kit (Tiangen, Beijing, China). Subsequently, cDNA synthesis was performed using the PrimeScript RT Kit (Toyobo, Shanghai, China), which served as a template for quantitative reverse transcription PCR (qRT-PCR). All work was performed in accordance with the manufacturer’s instructions. According to the nucleotide sequences of PDF family genes, eight primer pairs were designed using Primer 5.0 software (Table S1). GADPH was employed as the internal reference gene. The conditions and reaction system for qRT-PCR followed the protocol of SYBR PreMix Ex Taq™ II (Toyobo, Shanghai, China), with three biological replicates for each sample. The experimental data were obtained, and the expression levels were calculated using the 2^−∆∆CT^ method [68]. Finally, the results were normalized and presented in bar charts.

### 4.8. Prediction of the Protein Structures of MsPDF Genes

To better analyze the function of the *MsPDF* genes, the tertiary and quaternary structures of proteins encoded by the *MsPDF* genes were predicted using AF2 software (version 0.2.0). The quality of protein structure predictions was evaluated using the template modeling score (TM-score) [69]. Both the TM-score and the predictive template modeling score reflect whether the model accurately evaluates the results and they were positively correlated [70]. Therefore, the predictions were evaluated using the pTM score to assess prediction accuracy, and the ipTM score was used to evaluate the accuracy of subunit position predictions within the quaternary structure. The pLDDT score was used to assess the accuracy of the predicted model for residue-by-residue [71]. In addition, multiple sequence alignment (MSA) further enhanced the prediction accuracy for subunit positioning within the tetrameric structure [72]. The prediction result with the highest score was selected and visualized using the ChimeraX (version 1.9) software.

## 5. Conclusions

In this study, the MsPDF gene family was identified and characterized at the genome-wide level, with 11 members being classified into three subfamilies. Conserved motif analyses revealed seven motifs, which showed consistent sequential arrangements across all protein sequences. The promoter region of the *MsPDF* genes was significantly enriched for ARE, ABRE, and BOX 4 elements, which are essential for plant stress adaptation. They respond to abiotic stresses such as drought and salinity stress by participating in the abscisic acid (ABA) and ethylene signaling pathways. Integrated gene regulatory network and GO analyses revealed that the *MsPDF01*, *MsPDF03*, and *MsPDF10* interact with other alfalfa genes, which respond to abiotic stress by binding to metal ions. Furthermore, tissue-specific expression patterns of the *MsPDF* genes were observed under salinity stress, and qRT-PCR experiment verification confirmed their active participation in abiotic stress responses. Finally, the monomeric, dimeric, and tetrameric structures of the MsPDF protein were predicted. This study provides a foundation for illuminating the molecular mechanisms of *PDF* genes mediating abiotic stress adaptation in plants.

## Figures and Tables

**Table 1:** Summary of MsPDF gene family members identified in the alfalfa.

Name	Arabidopsis		Chromosomal Locations	Group	Intron	Length(aa)
	Homologous Gene					
	Gene	Gene				
	Accession NO.	Name				
*MsPDF01*	AT1G19610	*PDF1.4*	chr8.1:84174234-84174563	I	1	79
*MsPDF02*	AT1G55010	*PDF1.5*	chr8.1:83983356-83983809	I	1	79
*MsPDF03*	AT1G19610	*PDF1.4*	chr8.3:76391825-76392231	I	1	99
*MsPDF04*	AT1G19610	*PDF1.4*	chr8.3:76401833-76402163	I	1	79
*MsPDF05*	AT2G26010	*PDF1.3*	chr2.1:61335159-61335818	II	1	78
*MsPDF06*	AT1G61070	*PDF2.4*	chr2.3:18266357-18267529	II	1	73
*MsPDF07*	AT1G61070	*PDF2.4*	chr2.4:59797715-59798955	II	1	77
*MsPDF08*	AT5G63660	*PDF2.5*	chr3.2:81190189-81190502	III	1	73
*MsPDF09*	AT2G02100	*PDF2.2*	chr4.3:7552947-7553374	III	1	74
*MsPDF10*	AT2G02130	*PDF2.3*	chr8.4:32871559-32871973	III	1	76
*MsPDF11*	AT2G02100	*PDF2.2*	chr8.1:31454195-31454834	III	1	81

## Data Availability

The datasets presented in this study can be found in the Supplementary Materials.

## Supplementary Materials

The following supporting information can be downloaded at: https://www.mdpi.com/article/10.3390/plants14091312/s1. Table S1: Primers for the *MsPDF* genes; Figure S1: Composition of conserved motifs of the *MsPDF* genes; Table S2: The gene interaction network of the *MsPDF* genes.

## References

[1] Vriens K., Cammue B.P., Thevissen K. (2014). Antifungal plant defensins: Mechanisms of action and production. Molecules.

[2] Lay F.T., Anderson M.A. (2005). Defensins--components of the innate immune system in plants. Curr. Protein Pept. Sci..

[3] Fant F., Vranken W., Broekaert W., Borremans F. (1998). Determination of the three-dimensional solution structure of Raphanus sativus antifungal protein 1 by 1H NMR. J. Mol. Biol..

[4] Liu Y.J., Cheng C.S., Lai S.M., Hsu M.P., Chen C.S., Lyu P.C. (2006). Solution structure of the plant defensin VrD1 from mung bean and its possible role in insecticidal activity against bruchids. Proteins.

[5] Almeida M.S., Cabral K.M., Kurtenbach E., Almeida F.C., Valente A.P. (2002). Solution structure of Pisum sativum defensin 1 by high resolution NMR: Plant defensins, identical backbone with different mechanisms of action. J. Mol. Biol..

[6] García-Olmedo F., Molina A., Alamillo J.M., Rodríguez-Palenzuéla P. (1998). Plant defense peptides. Biopolymers.

[7] Zeya H.I., Spitznagel J.K. (1966). Cationic Proteins of Polymorphonuclear Leukocyte Lysosomes I. Resolution of Antibacterial and Enzymatic Activities. J. Bacteriol..

[8] Ganz T., Selsted M.E., Szklarek D., Harwig S.S., Daher K., Bainton D.F., Lehrer R.I. (1985). Defensins. Natural peptide antibiotics of human neutrophils. J. Clin. Investig..

[9] (1989). Insect immunity: Isolation from immune blood of the dipteran Phormia terranovae of two insect antibacterial peptides with sequence homology to rabbit lung macrophage bactericidal peptides. Proc. Natl. Acad. Sci. USA.

[10] Mendez E., Moreno A., Colilla F., Pelaez F., Limas G.G., Mendez R., Soriano F., Salinas M., de Haro C. (1990). Primary structure and inhibition of protein synthesis in eukaryotic cell-free system of a novel thionin, gamma-hordothionin, from barley endosperm. Eur. J. Biochem..

[11] Colilla F.J., Rocher A., Mendez E. (1990). gamma-Purothionins: Amino acid sequence of two polypeptides of a new family of thionins from wheat endosperm. FEBS Lett..

[12] Terras F.R., Schoofs H.M., De Bolle M.F., Van Leuven F., Rees S.B., Vanderleyden J., Cammue B.P., Broekaert W.F. (1992). Analysis of two novel classes of plant antifungal proteins from radish (*Raphanus sativus* L.) seeds. J. Biol. Chem..

[13] Carrasco L., Vázquez D., Hernández-Lucas C., Carbonero P., García-Olmedo F. (1981). Thionins: Plant peptides that modify membrane permeability in cultured mammalian cells. Eur. J. Biochem..

[14] Terras F.R., Eggermont K., Kovaleva V., Raikhel N.V., Osborn R.W., Kester A., Rees S.B., Torrekens S., Van Leuven F., Vanderleyden J. (1995). Small cysteine-rich antifungal proteins from radish: Their role in host defense. Plant Cell.

[15] Penninckx I.A., Eggermont K., Terras F.R., Thomma B.P., De Samblanx G.W., Buchala A., Métraux J.P., Manners J.M., Broekaert W.F. (1996). Pathogen-induced systemic activation of a plant defensin gene in Arabidopsis follows a salicylic acid-independent pathway. Plant Cell.

[16] Whitbred J.M., Schuler M.A. (2000). Molecular Characterization of CYP73A9 andCYP82A1 P450 Genes Involved in Plant Defense in Pea. Plant Physiol..

[17] Zhu-Salzman K., Salzman R.A., Ahn J.-E., Koiwa H. (2004). Transcriptional Regulation of Sorghum Defense Determinants against a Phloem-Feeding Aphid. Plant Physiol..

[18] Hanks J.N., Snyder A.K., Graham M.A., Shah R.K., Blaylock L.A., Harrison M.J., Shah D.M. (2005). Defensin gene family in Medicago truncatula: Structure, expression and induction by signal molecules. Plant Mol. Biol..

[19] Finkina E.I., Bogdanov I.V., Shevchenko O.V., Fateeva S.I., Ignatova A.A., Balandin S.V., Ovchinnikova T.V. (2024). Immunomodulatory Effects of the Tobacco Defensin NaD1. Antibiotics.

[20] Tantong S., Pringsulaka O., Weerawanich K., Meeprasert A., Rungrotmongkol T., Sarnthima R., Roytrakul S., Sirikantaramas S. (2016). Two novel antimicrobial defensins from rice identified by gene coexpression network analyses. Peptides.

[21] Kerenga B.K., McKenna J.A., Harvey P.J., Quimbar P., Garcia-Ceron D., Lay F.T., Phan T.K., Veneer P.K., Vasa S., Parisi K. (2019). Salt-Tolerant Antifungal and Antibacterial Activities of the Corn Defensin ZmD32. Front. Microbiol..

[22] Bártová V., Bárta J., Jarošová M. (2019). Antifungal and antimicrobial proteins and peptides of potato (*Solanum tuberosum* L.) tubers and their applications. Appl. Microbiol. Biotechnol..

[23] Du B., Haensch R., Alfarraj S., Rennenberg H. (2024). Strategies of plants to overcome abiotic and biotic stresses. Biol. Rev. Camb. Philos. Soc..

[24] Tang R., Tan H., Dai Y., Li L., Huang Y., Yao H., Cai Y., Yu G. (2023). Application of antimicrobial peptides in plant protection: Making use of the overlooked merits. Front. Plant Sci..

[25] Manzanares P., Giner-Llorca M., Marcos J.F., Garrigues S. (2024). Fighting pathogenic yeasts with plant defensins and anti-fungal proteins from fungi. Appl. Microbiol. Biotechnol..

[26] Deepthi V., Mohanakumar K.P., Rajamma U. (2023). Efficacy of defensins as neutralizing agents against the deadly SARS-CoV-2. J. Biomol. Struct. Dyn..

[27] Kushmerick C., de Souza Castro M., Santos Cruz J., Bloch C., Beirão P.S. (1998). Functional and structural features of gamma-zeathionins, a new class of sodium channel blockers. FEBS Lett..

[28] Spelbrink R.G., Dilmac N., Allen A., Smith T.J., Shah D.M., Hockerman G.H. (2004). Differential antifungal and calcium channel-blocking activity among structurally related plant defensins. Plant Physiol..

[29] Vriens K., Peigneur S., De Coninck B., Tytgat J., Cammue B.P.A., Thevissen K. (2016). The antifungal plant defensin AtPDF2.3 from Arabidopsis thaliana blocks potassium channels. Sci. Rep..

[30] Kovalchuk N., Li M., Wittek F., Reid N., Singh R., Shirley N., Ismagul A., Eliby S., Johnson A., Milligan A.S. (2010). Defensin promoters as potential tools for engineering disease resistance in cereal grains. Plant Biotechnol. J..

[31] Zhou Q., Luo D., Chai X., Wu Y., Wang Y., Nan Z., Yang Q., Liu W., Liu Z. (2018). Multiple Regulatory Networks Are Activated during Cold Stress in *Medicago sativa* L.. Int. J. Mol. Sci..

[32] Quan W., Liu X. (2024). Tandem mass tag (TMT)-based quantitative proteomics analysis reveals the different responses of contrasting alfalfa varieties to drought stress. BMC Genom..

[33] Xie J., Li Y., Jiang G., Sun H., Liu X., Han L. (2023). Seed color represents salt resistance of alfalfa seeds (*Medicago sativa* L.): Based on the analysis of germination characteristics, seedling growth and seed traits. Front. Plant Sci..

[34] Mäser P., Thomine S., Schroeder J.I., Ward J.M., Hirschi K., Sze H., Talke I.N., Amtmann A., Maathuis F.J., Sanders D. (2001). Phylogenetic relationships within cation transporter families of Arabidopsis. Plant Physiol..

[35] Dong Y., Wang Y., Tang M., Chen W., Chai Y., Wang W. (2023). Bioinformatic analysis of wheat defensin gene family and function verification of candidate genes. Front. Plant Sci..

[36] Giacomelli L., Nanni V., Lenzi L., Zhuang J., Dalla Serra M., Banfield M.J., Town C.D., Silverstein K.A., Baraldi E., Moser C. (2012). Identification and characterization of the defensin-like gene family of grapevine. Mol. Plant-Microbe Interact. MPMI.

[37] Lee S.C., Luan S. (2012). ABA signal transduction at the crossroad of biotic and abiotic stress responses. Plant Cell Environ..

[38] Chen D., Muhae-Ud-Din G., Liu T., Chen W., Liu C., Gao L. (2021). Wheat Varietal Response to Tilletia controversa J. G. Kühn Using qRT-PCR and Laser Confocal Microscopy. Genes.

[39] Krishnaswamy S.S., Srivastava S., Mohammadi M., Rahman M.H., Deyholos M.K., Kav N.N.V. (2008). Transcriptional profiling of pea ABR17 mediated changes in gene expression in Arabidopsis thaliana. BMC Plant Biol..

[40] Dai X., Xu Y., Ma Q., Xu W., Wang T., Xue Y., Chong K. (2007). Overexpression of an R1R2R3 MYB gene, OsMYB3R-2, increases tolerance to freezing, drought, and salt stress in transgenic Arabidopsis. Plant Physiol..

[41] Cheng M.C., Liao P.M., Kuo W.W., Lin T.P. (2013). The Arabidopsis ETHYLENE RESPONSE FACTOR1 regulates abiotic stress-responsive gene expression by binding to different cis-acting elements in response to different stress signals. Plant Physiol..

[42] Sharma A., Sidhu G.P.S., Araniti F., Bali A.S., Shahzad B., Tripathi D.K., Brestic M., Skalicky M., Landi M. (2020). The Role of Salicylic Acid in Plants Exposed to Heavy Metals. Molecules.

[43] Gu T., Qi Z., Wang Y., Chen S., Yan J., Qiu H., Yu Y., Fang Z., Wang J., Gong J. (2024). An endophytic fungus interacts with the defensin-like protein OsCAL1 to regulate cadmium allocation in rice. Mol. Plant.

[44] Kaur G., Tak Y., Asthir B. (2022). Salicylic acid: A key signal molecule ameliorating plant stresses. Cereal Res. Commun..

[45] Luo Y., Song Y. (2021). Mechanism of Antimicrobial Peptides: Antimicrobial, Anti-Inflammatory and Antibiofilm Activities. Int. J. Mol. Sci..

[46] Mostofa M.G., Rahman M.M., Ghosh T.K., Kabir A.H., Abdelrahman M., Rahman Khan M.A., Mochida K., Tran L.P. (2022). Potassium in plant physiological adaptation to abiotic stresses. Plant Physiol. Biochem. PPB.

[47] Tong T., Li Q., Jiang W., Chen G., Xue D., Deng F., Zeng F., Chen Z.H. (2021). Molecular Evolution of Calcium Signaling and Transport in Plant Adaptation to Abiotic Stress. Int. J. Mol. Sci..

[48] Yang T., Chaudhuri S., Yang L., Du L., Poovaiah B.W. (2010). A calcium/calmodulin-regulated member of the receptor-like kinase family confers cold tolerance in plants. J. Biol. Chem..

[49] Du Y.Y., Wang P.C., Chen J., Song C.P. (2008). Comprehensive functional analysis of the catalase gene family in Arabidopsis thaliana. J. Integr. Plant Biol..

[50] Yu W., Kong G., Ya H., He L., Wu Y., Zhang H. (2023). Comprehensive Analysis of the Catalase (CAT) Gene Family and Expression Patterns in Rubber Tree (*Hevea brasiliensis*) under Various Abiotic Stresses and Multiple Hormone Treatments. Int. J. Mol. Sci..

[51] Wei Y., Chen H., Wang L., Zhao Q., Wang D., Zhang T. (2022). Cold acclimation alleviates cold stress-induced PSII inhibition and oxidative damage in tobacco leaves. Plant Signal. Behav..

[52] Singh V., Hallan V., Pati P.K. (2024). Withania somnifera osmotin (WsOsm) confers stress tolerance in tobacco and establishes novel interactions with the defensin protein (WsDF). Physiol. Plant..

[53] Wang S., Liu Y., Hao X., Chen Y., Wang Z., Shen Y. (2024). Enhancing plant defensins in a desert shrub: Exploring a regulatory pathway of AnWRKY29. Int. J. Biol. Macromol..

[54] Wu Y.R., Lin Y.C., Chuang H.W. (2016). Laminarin modulates the chloroplast antioxidant system to enhance abiotic stress tolerance partially through the regulation of the defensin-like gene expression. Plant Sci. Int. J. Exp. Plant Biol..

[55] Meng L., Feng Y., Zhao M., Jang T., Bi H., Ai X. (2024). Hydrogen peroxide mediates melatonin-induced chilling tolerance in cucumber seedlings. Plant Cell Rep..

[56] Chen H., Zeng Y., Yang Y., Huang L., Tang B., Zhang H., Hao F., Liu W., Li Y., Liu Y. (2020). Allele-aware chromosome-level genome assembly and efficient transgene-free genome editing for the autotetraploid cultivated alfalfa. Nat. Commun..

[57] Finn R.D., Coggill P., Eberhardt R.Y., Eddy S.R., Mistry J., Mitchell A.L., Potter S.C., Punta M., Qureshi M., Sangrador-Vegas A. (2016). The Pfam protein families database: Towards a more sustainable future. Nucleic Acids Res..

[58] Edgar R.C. (2004). MUSCLE: Multiple sequence alignment with high accuracy and high throughput. Nucleic Acids Res..

[59] Saitou N., Nei M. (1987). The neighbor-joining method: A new method for reconstructing phylogenetic trees. Mol. Biol. Evol..

[60] Minh B.Q., Nguyen M.A., von Haeseler A. (2013). Ultrafast approximation for phylogenetic bootstrap. Mol. Biol. Evol..

[61] Bailey T.L., Boden M., Buske F.A., Frith M., Grant C.E., Clementi L., Ren J., Li W.W., Noble W.S. (2009). MEME SUITE: Tools for motif discovery and searching. Nucleic Acids Res..

[62] Chen C., Wu Y., Li J., Wang X., Zeng Z., Xu J., Liu Y., Feng J., Chen H., He Y. (2023). TBtools-II: A “one for all, all for one” bioinformatics platform for biological big-data mining. Mol. Plant.

[63] Lee T., Yang S., Kim E., Ko Y., Hwang S., Shin J., Shim J.E., Shim H., Kim H., Kim C. (2015). AraNet v2: An improved database of co-functional gene networks for the study of Arabidopsis thaliana and 27 other nonmodel plant species. Nucleic Acids Res..

[64] Doncheva N.T., Morris J.H., Gorodkin J., Jensen L.J. (2019). Cytoscape StringApp: Network Analysis and Visualization of Proteomics Data. J. Proteome Res..

[65] Alexa A., Rahnenführer J., Lengauer T. (2006). Improved scoring of functional groups from gene expression data by decorrelating GO graph structure. Bioinformatics.

[66] Zhang X., Yang H., Li M., Bai Y., Chen C., Guo D., Guo C., Shu Y. (2022). A Pan-Transcriptome Analysis Indicates Efficient Downregulation of the FIB Genes Plays a Critical Role in the Response of Alfalfa to Cold Stress. Plants.

[67] Medina C.A., Samac D.A., Yu L.X. (2021). Pan-transcriptome identifying master genes and regulation network in response to drought and salt stresses in Alfalfa (*Medicago sativa* L.). Sci. Rep..

[68] Livak K.J., Schmittgen T.D. (2001). Analysis of Relative Gene Expression Data Using Real-Time Quantitative PCR and the 2^−ΔΔCT^ Method. Methods.

[69] Zhang Y., Skolnick J. (2004). Scoring function for automated assessment of protein structure template quality. Proteins.

[70] Jumper J., Evans R., Pritzel A., Green T., Figurnov M., Ronneberger O., Tunyasuvunakool K., Bates R., Žídek A., Potapenko A. (2021). Highly accurate protein structure prediction with AlphaFold. Nature.

[71] Ma P., Li D.W., Brüschweiler R. (2023). Predicting protein flexibility with AlphaFold. Proteins.

[72] Bryant P., Noé F. (2024). Improved protein complex prediction with AlphaFold-multimer by denoising the MSA profile. PLoS Comput. Biol..

