# Supplementary material for: Identification of the Plant Defensin (MsPDF) Gene Family in Medicago sativa and Analysis of Expression Patterns Under Abiotic Stress
Source: Plants (Basel). 2025 Apr 26;14(9):1312. doi: 10.3390/plants14091312 (PMC12073698; doi:10.3390/plants14091312)
Supplement: Supplementary file 1 [file plants-14-01312-s001.zip › Table S2.pdf]

Table S2. The gene interaction network of the *MsPDF* genes

MsPDF genes	Other genes in *Medicago sativa*
MsPDF01	*MS.gene068294*
	MS.gene97592
	MS.gene34288
	MS.gene065173
	MS.gene06022
	MS.gene047547
	MS.gene67016
	MS.gene63098
	MS.gene043044
	MS.gene072475
	MS.gene55766
	MS.gene90092
	MS.gene87282
	MS.gene80162
	MS.gene72897
	MS.gene71655
	MS.gene60497
	MS.gene60100
	MS.gene56628
	MS.gene55764
	MS.gene47524
	MS.gene41022
	MS.gene37676
	MS.gene34604
	MS.gene33455
	MS.gene31081
	MS.gene31045
	MS.gene30366
	MS.gene26914
	MS.gene25879
	MS.gene24357
	MS.gene072912
	MS.gene071221
	MS.gene070060
	MS.gene068779
	MS.gene068320
	MS.gene066491
	MS.gene057717
	MS.gene048949
	MS.gene047675
	MS.gene04593
	MS.gene038936
	MS.gene037397
	MS.gene023963

	MS.gene005253
	MS.gene002058
	MS.gene014308
MsPDF03	*MS.gene019951*
	MS.gene00816
	MS.gene072475
	MS.gene043044
	MS.gene002058
	MS.gene068294
	MS.gene047547
	MS.gene67016
	MS.gene34288
	MS.gene065173
	MS.gene037092
	MS.gene55766
	MS.gene005253
	MS.gene06022
	MS.gene63098
	MS.gene84662
	MS.gene77909
MsPDF10	*MS.gene66736*
	MS.gene50204
	MS.gene35122
	MS.gene34032
	MS.gene28100
	MS.gene060595
	MS.gene037097
	MS.gene030443
	MS.gene01685
	MS.gene016799
	MS.gene014308
	MS.gene009450
	MS.gene006796
	MS.gene002964
	MS.gene97592
	MsPDF01
