# Supplementary material for: Identification of the Plant Defensin (MsPDF) Gene Family in Medicago sativa and Analysis of Expression Patterns Under Abiotic Stress
Source: Plants (Basel). 2025 Apr 26;14(9):1312. doi: 10.3390/plants14091312 (PMC12073698; doi:10.3390/plants14091312)
Supplement: Supplementary file 1 [file plants-14-01312-s001.zip › Table S1.pdf]

Table S1. Primers for the MsPDF gene

Name	Forward primer sequences (5'→3')	Reverse primer sequences (5'→3')
MsPDF02	GGCTTCCTCTTCTCCTAAATTC	ATGCAAGTCCCGCCAA
MsPDF03	GCCTTGCCTTTCTCTTGATT	TTTGGAGCGCATTTTCTGTA
MsPDF04	CCTCTGCTAATAAATTTTACACCAT	TGACAAGCACCAAAAGTAGC
MsPDF05	TGGAGAAGAAATCAGTAGCTAGA	CACTAATTGCGTGCTCTTTG
MsPDF08	ATGGAGAGGAAATCACTTGTC	GCATCTGCCACCAGAGAA
MsPDF09	GAGGAAAACACTCGGCATTT	CGACAATCACCACCAATGAA
MsPDF10	TCGTTCAGTTCCTTTGGTTT	GCAGTGTCCTCCAGAGAAA
MsPDF11	CGTCCAATCCCTTCAAATCG	AATCTCCTCCTGTGAAACCC
