# Supplementary figures and images for: Identification of the Plant Defensin (MsPDF) Gene Family in Medicago sativa and Analysis of Expression Patterns Under Abiotic Stress
Source: Plants (Basel). 2025 Apr 26;14(9):1312. doi: 10.3390/plants14091312 (PMC12073698; doi:10.3390/plants14091312)

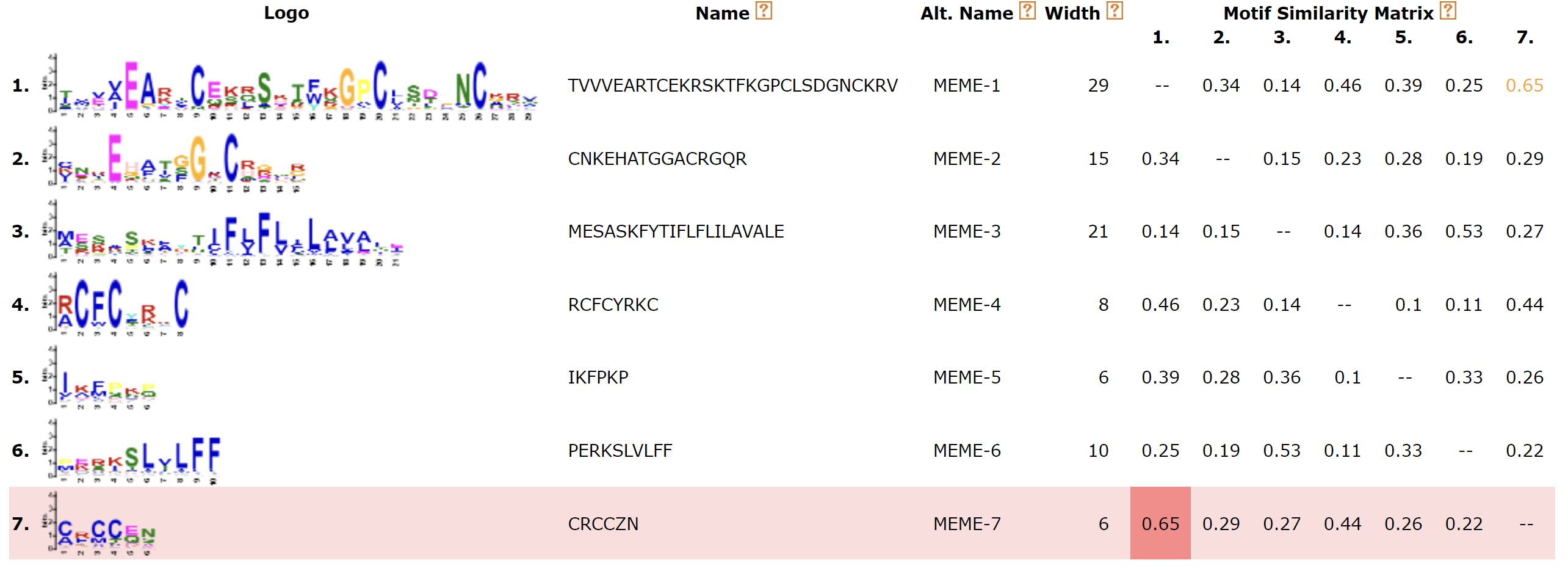

Supplement: Supplementary file 1 [file plants-14-01312-s001.zip › Figuer S1.jpg]
